# Recruitment, retention and reporting of ethnic representativeness in maternity trials: a scoping review

**DOI:** 10.1136/bmjopen-2025-098926

**Published:** 2025-11-26

**Authors:** Holly Lovell, Emilie Wicks, Hilary Thompson, Leanna Brace, Tomasina Stacey, Hannah Rayment-Jones, Seeromanie Harding

**Affiliations:** 1 Population Health Sciences, King’s College London, London, UK; 2 Women’s Services, Guy’s and St Thomas’ Hospitals NHS Trust, London, UK; 3 Methodologies, King’s College London, London, UK; 4 Women and Children’s Health, King’s College London, London, UK; 5 Population Health Sciences, King’s College London Faculty of Life Sciences and Medicine, London, UK

**Keywords:** Clinical Trial, Pregnancy, OBSTETRICS, Postpartum Period, Health Equity

## Abstract

**Abstract:**

**Introduction:**

Black and Asian women experience significantly higher rates of mortality and morbidity perinatally compared with white women and are more likely to lose their babies. These groups are also under-represented in clinical research, resulting in evidence that may not be generalisable. Tools have been developed to facilitate the inclusion of ethnic minority groups, but it is unknown to what extent representation and inclusion are considered in maternity trials.

**Aim:**

To provide an overview of how ethnically diverse recruitment is considered and reported in maternity trials in the UK.

**Methods:**

A scoping review was conducted, undertaking a systematic search to identify published trial protocols and their subsequent results papers, conducted within the UK, recruiting women during pregnancy or within 6 weeks postnatally between 2004 and 2024.

Data was extracted from protocols on whether representation of participants was considered in the study design and if specific recruitment and retention strategies were planned for ethnic minority groups.

Data extracted from results papers identified whether representation of participants was discussed and if recruitment strategies were discussed; these were compared against the protocol.

**Results:**

A total of 96 published protocols met the inclusion criteria; 8 mentioned specific recruitment strategies and 5 mentioned specific retention strategies. Only two included both recruitment and retention strategies. The most common strategies included providing different types of language support and adapting interventions to be culturally appropriate. Strategies were not evaluated.

67 results papers were available. Ethnicity was reported in 57 papers, with heterogeneity of categories between papers. Only 32 papers discussed representativeness of participants.

**Conclusion:**

Few maternity trials report considerations on how they ensure they are recruiting and retaining ethnically representative participants. Minimal discussion is undertaken around the extent to which trial participants reflect the population to which findings will be applied.

Further work is needed to support implementation and evaluation of inclusive research guidance. Failing to ensure those from ethnic minority groups are included in research can exacerbate inequalities.

**STRENGTHS AND LIMITATIONS OF THIS STUDY**

- Reviewing published trial protocols alongside their results papers is an innovative way to provide a detailed insight into how inclusion and ethnic representativeness is considered across the research cycle.
- Including trials which focused on the general maternity population, rather than ethnic minority groups alone, provided a broader view on how inclusion is considered across perinatal research.
- Due to limits placed on publications, it may be that inclusive strategies were used by researchers which were not reported within the protocols.
- The review focused on published protocols and therefore may not represent other perinatal trials which published study findings only.

## Background

 Significant ethnic inequalities in maternity outcomes exist.^1^,^4^ The confidential enquiries into maternal and perinatal deaths in the UK have consistently demonstrated a higher mortality rate for Black and Asian women and babies, and migrants born outside the UK, compared with white women.^24^,^9^ Furthermore, those from ethnic minority groups experience higher rates of morbidity, including increased risks of prematurity, gestational diabetes and complications related to hypertension in pregnancy.^10^,^13^

In this paper, the current guidance from the UK government on writing about ethnicity is used. The term ‘ethnic minority’ refers to all groups except white British.^14^ However, the authors acknowledge the complex construct of ethnicity, which must be considered when discussing ethnic inequalities. Although ethnicity encompasses some physical features, groups are also socially defined by a shared geographical heritage, beliefs, behaviours and culture.^15^ Consequently, we should not make causative statements implying ethnicity is a biological reason for inequalities.^16 17^ Caution in reporting ethnicity in these ways has been recognised in epidemiological research for over 20 years^18^ as grouping people in broad categories neglects the vast heterogeneity within groups.

Ethnic health inequalities are shaped by multifactorial influences, often explained through the structural determinants of health such as housing, education and employment.^19^ Area deprivation, an ecological correlate of these determinants, is strongly associated with adverse maternal outcomes; women and babies in the most deprived areas face the highest mortality risks.^8^ Recent evidence from a UK national population-based cohort study showed that women of black ethnicity remained at significantly increased risk of maternal death compared with women of white ethnicity after adjustments for area deprivation.^20^ Reviews have also highlighted that persistent inequalities reflect the impact of structural racism, implicit bias and discriminatory practices within maternity care.^21^,^28^ Under-representation of ethnic minority groups in research, inadequate recognition of cultural and linguistic needs, and reliance on Eurocentric models of care can further exacerbate these disparities, perpetuating mistrust, disengagement and poorer health outcomes for minority groups.

An intersectional perspective is essential to fully interrogate these inequalities. Ethnicity does not operate in isolation but intersects with gender, class, migration status and other axes of structural disadvantage to shape women’s experiences of maternity care.^29 30^ Ignoring these intersections risks oversimplifying the mechanisms driving inequality and overlooking groups who experience multiple disadvantage.^31 32^ Addressing ethnic health inequalities therefore requires structural reform: embedding accountability for equity within healthcare systems, developing culturally safe and inclusive models of care and diversifying research participation to ensure the complexity of intersecting inequalities is adequately captured.

Ensuring evidence incorporated into clinical guidelines is representative of those it will be applied to is vital.^33 34^ Yet participants from Black, Asian and ethnic minority groups are often under-represented,^35^,^37^ which can impact the reliability and generalisability of research. Cultural beliefs and behaviours can affect responses to, and acceptability of both medical and behavioural interventions.^37^ In addition, ethnic minority populations may experience multiple and cumulative risk exposures related to racism and discrimination that can result in biological changes, potentially impacting the effectiveness of treatment interventions.^38 39^

In recognising the implication of under-representation, guidance and toolkits have been created to facilitate researchers in the design of inclusive research. Advice includes undertaking meaningful patient and public involvement and engagement (PPIE) from the inception of research,^34 40 41^ alongside identifying the demographics of the population who should be represented and designing targeted recruitment and retention strategies accordingly.^3537 42^,^44^ Representation can often be difficult to assess, and it is dependent on factors including where the research was conducted, the condition under examination and who the results will be applied to. Therefore, the term ‘appropriate representation” may be more useful when describing representation to acknowledge that comparing study populations to the general population is not always suitable.

The available recommendations are not mandated and have not been evaluated. Therefore, it is unknown the extent to which they are used, how many researchers are aware of them or what their impact is.

It is unknown whether maternity trials incorporate guidance to improve the inclusion of ethnically diverse and representative participants, and if strategies are being employed, whether their effectiveness is being evaluated.

### Aim

This scoping review aims to provide an overview of how maternity trials in the UK report plans for the recruitment and retention of participants from ethnic minority groups, and how the representation of these groups is considered and reported. This will highlight examples of good practice and recommendations for improvements.

### Review questions

- How often are specific strategies being reported to recruit and retain ethnically diverse participants?
- What strategies are being used to recruit and retain ethnically diverse participants?
- Are strategies evaluated? How? And what are their findings?
- Is representation by ethnicity of participants considered when discussing findings of maternity research?
- Is intersectionality considered within protocols or trial findings?

## Method

The review followed a scoping review methodology conducted in accordance with the Joanna Briggs Institute (JBI) guidelines^45^ and is reported as per Preferred Reporting Items for Systematic reviews and Meta-Analysis extension for Scoping Reviews (PRISMA-ScR) guidelines.^46^ The protocol is registered on the Open Science Framework (available at: https://osf.io/hs2bg/?view_only=78f4b0b141b74ef697b53d9b3ea35e0b). A scoping review method was chosen as it is the recommended approach when exploring how research is being conducted and when providing an overview of the literature.^47 48^

### Types of sources

The review focused on published protocols, as compared with results papers they have more scope to describe recruitment methods. Available papers reporting results of randomised controlled trials (RCTs) of the included protocols that met the inclusion criteria were also included. These were compared and examined together to give an overview of whether targeted approaches were used and evaluated, and whether this was reflected in the published demographics of participants. A 20-year period was searched, as representation in research is a relatively recent priority, therefore it was unlikely older research would include detailed considerations on the topic.

### Eligibility criteria

As per the JBI methodology, a search strategy was developed using a Participants, Concept, Context approach.

#### Participants

Women and birthing people who are pregnant (any gestation) or up to 6 weeks postnatal.

#### Concept

The inclusion, recruitment and retention of participants from diverse and appropriately representative ethnic backgrounds in maternity research trials.

#### Context

Maternity RCTs conducted within the UK.

Healthcare guidelines prioritise the inclusion of evidence from RCTs^49^ and these are considered the gold standard in evidence-based practice. Therefore, this review only included maternity research that used an RCT design.

#### Inclusion criteria

- Published between 1 January 2004 and 13 December 2024.
- Recruitment undertaken in the UK.
- Studies recruiting participants during pregnancy or the first 6 weeks postnatally.
- Protocol of a RCT study design.

#### Exclusion criteria

- Studies not recruiting human participants.
- Research undertaking analysis of routinely collected data.
- Observational studies, case control studies and cohort studies.
- Full text paper unavailable in the English language.

### Search strategy

A systematic search of EMBASE, Medline, PsycINFO and CINAHL was conducted to identify published protocols. Key terms were defined based on Participants, Concept and Context and the inclusion criteria. Search terms also incorporated previously validated search filters for pregnancy,^50^ the UK^51^ and RCTs^52^ (online supplemental file 1).

Subsequent results papers of the included protocols were identified through searching Google Scholar and clinical trials registries. The search was completed by 13 December 2024.

### Screening and evidence selection

Search results were uploaded into Covidence,^53^ an online software that supports the management of systematic reviews. Duplicates were removed and two independent reviewers screened titles and abstracts against the inclusion criteria. The full text papers of those that met the inclusion criteria were retrieved and imported into Covidence for further assessment, conducted independently by two reviewers. The reviewers resolved any disagreements through discussion. Reasons for exclusion of full text papers are reported with the reasons recorded.

### Data extraction

Data were extracted using two tools developed for the review (online supplemental file 2). Two reviewers independently piloted and tested the tools across four sources to check for reliability. Two reviewers independently extracted data from the protocols. The first reviewer extracted data from the results papers, which was then verified by a second reviewer. Discrepancies were resolved through discussion or through input from another reviewer. Recruitment and retention strategies were broadly defined, to include any research design consideration that specifically referred to consideration of ethnic minority populations, inclusion or representation. Extracted statements could range from a single sentence to a broader description, for example, a protocol may state information is translated to increase participation of those from ethnic minorities. Identification was based on the authors’ expertise in trial conduct and knowledge of recommendations from inclusion frameworks, for example, the National Institute for Health and Care Research (NIHR) INCLUDE ethnicity framework recommends researchers identify which populations should be included in the trial, and then designing targeted recruitment methods to ensure they are accessible to those groups.^37^

### Data analysis

The authors charted findings against the review questions and undertook a descriptive synthesis. They collated identified strategies thematically and compared with available results papers to identify if proposed targeted strategies were used as planned, and what the consequences of these were. The authors did not conduct quality appraisals, as quality of the overall paper was not an aim of the review.^1^

### Patient and public involvement

There has been no patient or public involvement in this review.

### Ethical statement

Ethical approval was not required for this review.

## Results

**Figure 1:**
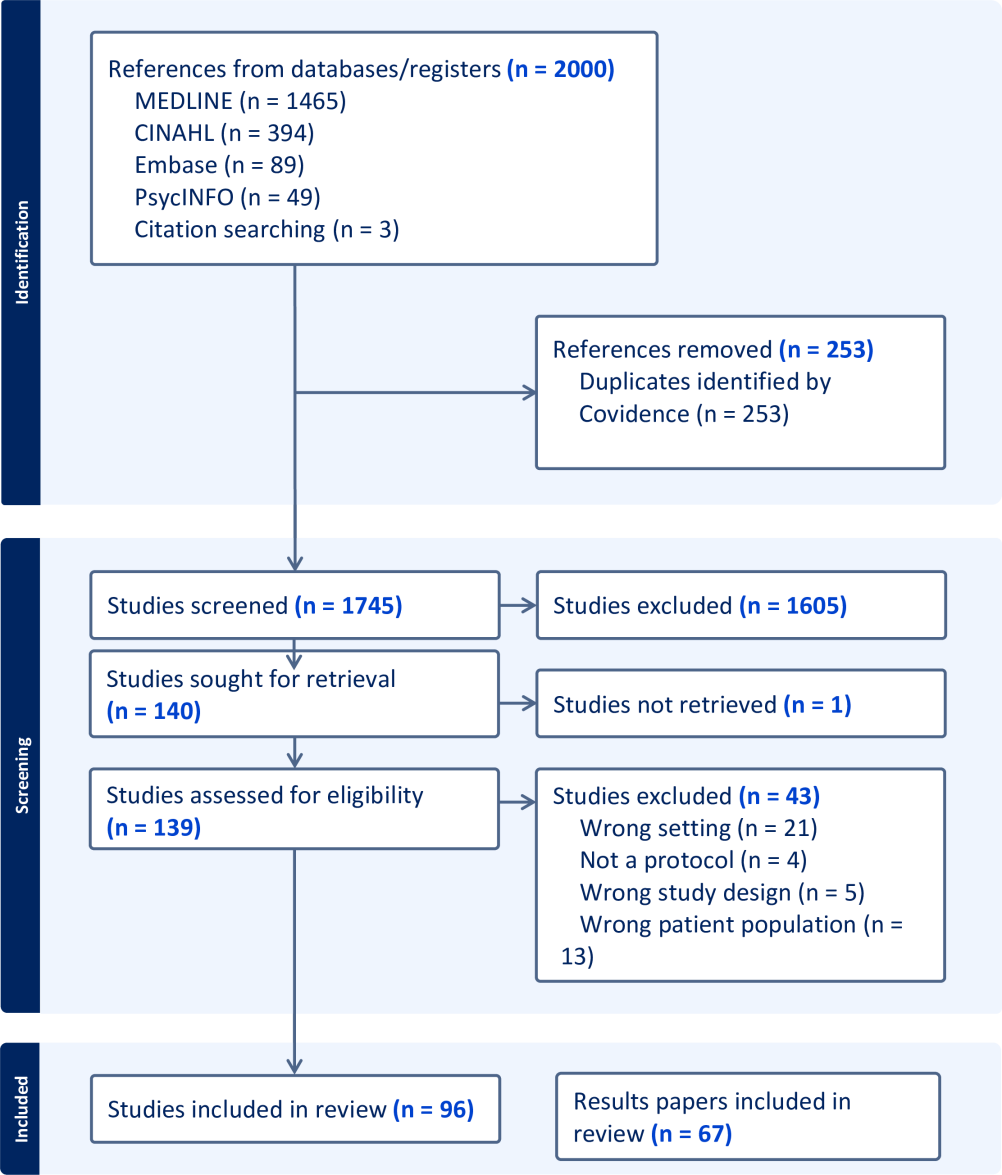
PRISMA-ScR flow diagram. PRISMA-ScR, Systematic reviews and Meta-Analysis extension for Scoping Reviews.

After title and abstract screening, 140 published protocols met the inclusion criteria and 96 protocols remained after full text review. Of these, 67 had published results papers; the remaining trials were either ongoing or had recently completed and not yet published (PRISMA-ScR flow diagram figure 1). Results are presented by each review question. Table 1 provides an overview of the numbers of protocols and results papers which reported on each question.

**Table 1:** Summary of papers included per review question

Protocols	Number of protocols (N=96) which included
Recruitment strategy for ethnic minority participants	8
Retention strategy for ethnic minority participants	5
Involvement of PPIE in any recruitment or retention strategies	11
Plans to evaluate recruitment or retention strategies	6
Plans to assess representation of participants	4

RCT results papers	Number of papers (N=67) which included
Discussion of representation of participants	32
Ethnicity of participants reported	57
Description of how ethnicity was determined	11
Intersectionality discussed in demographics of participants or findings	0

PPIE, patient and public involvement and engagement; RCT, randomised controlled trial.

### How often are specific strategies being used to recruit and retain ethnically diverse participants?

The majority (85/96) of protocols did not explicitly specify a recruitment or retention strategy for participants from ethnic minority groups. Eight out of 96 protocols included a recruitment strategy for ethnic minority participants^54^,^61^ and 5 protocols included a retention strategy.^5657 62^,^64^ Two included both a recruitment and retention strategy.^56 57^ The characteristics of these 11 protocols and their complete data extraction tables are available in online supplemental file 3.

### What strategies were used to recruit and retain ethnically diverse participants?

Strategies to improve inclusion of participants from ethnic minority backgrounds were mostly brief references rather than detailed plans. Table 2 illustrates the identified strategies and the protocols in which they were included. Seven of the eight protocols that considered recruitment of ethnic minority participants stated an interpreter would be used if required for recruitment;^54^,^5658^ however, only two planned for translated participant information sheets.^55 59^ Davies *et al* also stated a recruitment video, with a preferred language voiceover or subtitles, would be available.^55^

**Table 2:** Reported recruitment and retention strategies

Strategies	Recruitment					Recruitment and retention	Retention	
	Use of interpreters	Translation of participant information sheets	Choosing trial sites with high levels of diversity	Training research and clinical staff in inclusive recruitment	Recruitment via social media and community organisations	Bilingual health advocates	Consideration of culture and socioeconomic circumstances in intervention design	Use of interpreters
Protocol and intervention								
Harvey *et al*^59^ 1000 IU cholecalciferol orally daily in pregnant women from 14 weeks	X	X						
Al Watter *et al*^64^ A simple, targeted Mediterranean-based diet, supplemented with extra virgin olive oil and nuts							X	
Hezelgrave *et al*^54^ A cervical cerclage versus a cervical pessary versus vaginal progesterone	X							
Wiggins *et al*^57^ A group model of antenatal care (Pregnancy Circles)	X					X		
Bick *et al*^62^ Standard care, plus information on positive lifestyle behaviours from late pregnancy and access to a postnatal 12-week weight management group							X	
Chappell *et al*^58^ Planned delivery with minimal delay with initiation of delivery within 48 hours of randomisation	X							
Hurrell *et al*^60^ Repeat revealed PlGF-based testing compared with repeat concealed testing, for suspected preterm pre-eclampsia	X							
Wiggins *et al*^56^ A group model of antenatal care (Pregnancy Circles)	X		X	X				X
Davies *et al*^55^ Monitoring maternal glucose levels using a CGM device (Freestyle Libre2) and smartphone/reader	X	X						
McAuley *et al*^63^ A pragmatic lifestyle education intervention for weight reduction during and post pregnancy							X	
Volkmer *et al*^61^ 12 web-based Cognitive Bias Modification for Interpretation training sessions in a 4-week period.					X			

CGM, continuous glucose monitoring.

Widening recruitment beyond the hospital site was planned by Volkmer *et al* to facilitate recruitment of those from Black and Asian backgrounds. A strategy was developed to share study information using social media with endorsements from their charity and community partners.^61^

Fewer retention strategies were referred to. Addressing language needs was again highlighted, either through an interpreter^56^ or a bilingual health advocate.^57^ Three papers stated they considered cultural needs and socioeconomic circumstances in the design of their interventions, which may support retention of ethnic minority participants.^62^,^64^ This included integrating cultural preferences when giving dietary advice in a trial on postnatal weight management in pregnancy^62^ and culturally adaptable recipes in a trial examining the impact of a Mediterranean style diet on outcomes for women with metabolic risk factors.^64^

The two protocols that included both recruitment and retention strategies provided more detailed plans^56 57^ and were a pilot study followed by a full trial from the same research group. The pilot study focused on language diversity within their study population, a key consideration throughout their study design, although it was not specified which languages. The use of ‘bilingual health advocates’ was planned to work alongside the researcher undertaking recruitment and throughout all other study procedures. Advocates were from an external agency or via the NHS Trust and research-specific training was provided. However, in the full trial, the researchers stated plans for language support would only be through interpreters booked via the NHS Trusts, researchers who spoke the required languages, phone interpreting services or using informal support from friends or family.^56^ The full trial also planned to select trial sites in areas of high ethnic diversity, although it was not stated how this was defined. Training for recruiters and midwives on how to improve inclusive recruitment practices, including not making assumptions about possible recruits, was also planned.^56^

No additional recruitment or retention strategies were identified in results papers that were not addressed in their protocols.

#### Involvement of PPIE in recruitment or retention strategies

35 protocols referred to input from PPIE members in overall study design. References were often non-specific; however, 11 protocols stated PPIE members either contributed to the design of recruitment pathways, identified potential barriers to participation or were involved in troubleshooting recruitment and retention issues once the trial had commenced.^5761 62 65^,^72^ Demographic details on PPIE members were not reported, therefore it is unknown whether those involved were representative of the target population.

### If strategies were evaluated, how were they evaluated and what were the outcomes?

Only two protocols that included specific recruitment or retention plans for ethnic minority participants stated plans to evaluate their strategies. Plans to assess recruitment or retention challenges through a short interview and questionnaire for women who declined, interviews with participants and also those who left the intervention were stated in a feasibility trial.^57^ Yet in the subsequent results paper, this evaluation did not refer to ethnic minority groups.^73^ Plans for the follow-up full trial also included plans to evaluate retention; however, not specific to ethnic minority participants or in relation to their planned recruitment and retention strategies.^56^

Of the protocols that included specific recruitment or retention strategies for ethnic minority participants, seven had published results papers. Six appeared to have successfully recruited ethnically diverse samples with non-white participants representing between 29% and 62% of participants (see online supplemental file 3 for further details). Although strategies were not evaluated, it is possible this contributed to their recruitment.

An additional four protocols included plans to evaluate their recruitment or retention strategies, including exploration around the impact of ethnicity, through a range of qualitative methods.^55 69 74 75^ One paper has since published their trial results, and the only reference related to the impact of ethnicity on recruitment or retention was an interview extract from a research midwife who felt the intervention, a natural vegetarian supplement, encouraged recruitment as it would appeal to the largely Muslim population.^76^

### Is the representation of participants considered when discussing findings of maternity research?

Four protocols included plans to assess representation of participants.^7477^,^79^ Two planned to compare baseline demographics data, including ethnicity, with the wider population to assess generalisability,^77 78^ yet in their subsequent results papers, this was not reported or discussed.^80 81^ Three protocols proposed they would explore the effects of demographics on recruitment and retention rates,^74 77 79^ but this was not reported in the available results papers.^76 81^

32 of the 67 results papers discussed representation of participants, with 11 papers recognising ethnic minority groups were under-represented.^82^,^92^ Studies that reported they had recruited representative samples generally made broad statements, declaring samples were representative of the wider population or those most at risk of the condition under examination, or that they had included a broad range of sociodemographic variables.^7376 93^,^99^ Yet these statements were not supported by data to determine how they had reached these conclusions. One trial recruiting type 1 diabetics in pregnancy provided an exemplar example demonstrating the representation of their participants through inclusion of a supplementary table presenting evidence of the demographics of the type 1 diabetic population in the UK compared with their study population.^100^

### Recording of ethnicity and demographics

Ethnicity was reported in 57 of the 67 results papers. The majority used ethnic groups based on the five top level categories used by the Office for National Statistics (ONS)^101^; White, Black, Asian, Mixed, Other. 11 papers reported directly comparable ethnic categories, with variation across the remaining 46 papers. Papers that reported ethnicity included a total of 64 different ethnic categories.

11 papers included a description of how ethnicity was determined. 10 stated ethnicity was self-reported, and 1 reported ethnicity was determined as coded by the National Health Service (NHS), but it was not specified where this was retrieved from.^88^ Four papers did not specify whether self-reporting was chosen from a list of predetermined categories or free text;^7383 102^,^104^ however, the remaining six stated ethnicity was chosen from options based on ONS categories.^8498 105^,^107^

The papers reported a variety of other demographics, including age, index of multiple deprivation quintile, highest education, employment and living arrangements. Authors mainly used ethnicity and other demographics to measure homogeneity between trial arms. Outcomes by ethnicity were only reported in 6 of the 65 results papers,^86 90 91 98 107 108^ with only one including this within a table in the main text.^98^

### Intersectionality

Neither protocols nor their subsequent results papers discussed intersectionality and its potential implications around recruitment of participants, impact on findings or representative samples.

## Discussion

This scoping review suggests limited approaches to recruiting and retaining ethnic minority populations are planned in maternity trials. A total of 11 protocols specified targeted strategies, with 8 protocols specifying recruitment strategies and 5 suggesting retention strategies, 2 of which included both. In addition, considerations around representation of ethnic minority participants and the subsequent implications for generalisability were not routinely included in discussions.

Failing to ensure those who experience the poorest outcomes are included in research can exacerbate inequalities. This is due to reduced generalisability of trials, missing differences in responses to interventions, fuelling lack of trust. The role of under-representation in research perpetuating inequalities has also been demonstrated to have an economic impact.^109^

A range of reasons for under-representation of ethnic minority groups in research is discussed in the literature. Barriers to participation include restrictive study designs, poor engagement with communities and a history of mistrust, with most evidence from the USA and the UK and no substantial differences reported between the two countries.^35 37 110 111^ To address these issues, a range of recommendations has been published to support the inclusion of ethnic minority participants across the research cycle.^3537 42^,^44^ These include identification of who might be under-represented within the researcher’s population of interest and who experiences higher burden of disease, and then exploring potential barriers to their inclusion and how they might be mitigated.^43^ This may involve designing different formats and methods of sharing study information and working with communities to identify appropriate and flexible recruitment pathways.^35 37 44 111^ No protocols provided substantive discussions that suggested the demographics of who should be included had been considered at the design stage and incorporated into recruitment strategies. How to determine proportionate representation can be difficult; however, it must still be considered, and guidance published this year by the STRIDE group includes recommendations to support these decisions.^33^ It may be argued that including a subgroup analysis by ethnic group is an indicator of consideration of representation. Although six papers did include this, it was not discussed whether sample sizes were powered for this to be significant. Therefore, subgroup analysis in the absence of discussion around adequately powered sample sizes for each group is not an indication of representation.

Despite available guidance, only 2 of the 11 protocols that considered specific recruitment or retention strategies detailed comprehensive approaches,^56 57^ the remaining papers providing a brief reference to considerations made. These were often single sentences stating, for example, that sites with diverse populations would be chosen, or that an interpreter would be used where required. Protocols which reported more detailed considerations gave descriptions as to the different ways language needs would be supported, alongside discussion as to why inclusive approaches were important.

Strategies mainly focused on addressing language barriers, an often-cited barrier to research participation.^112^ Yet the use of interpreters or translated materials alone may neglect additional issues associated with language, such as health literacy which may impede understanding of what clinical research is.^113^ In addition, when undertaking translation of study documents, robust methods must be used, such as forwards and backwards translation to ensure accuracy;^113^ however, information on the processes used was not included in the protocols.

Researchers’ unconscious biases, alongside a lack of cultural awareness and an understanding of the historical mistrust surrounding research, have been highlighted in reviews of the literature as a barrier to recruitment. To address this, training is recommended.^34 111 112^ Yet only one trial referred to inclusive recruitment training for staff.^56^

The design of interventions should be culturally appropriate to support recruitment and retention;^37^ however, only three trials incorporated these considerations.^62^,^64^ It may be that some interventions lend themselves to being more culturally adaptable than others, as these trials were testing either a behavioural or dietary intervention, as opposed to medication or a device. This reflects that some recommendations are more easily adopted by certain trials, and it is not necessarily a reflection of lack of consideration.

Variety in recruitment locations may also be more appropriate for only certain trial interventions. Most trials planned to recruit from NHS settings, with only one trial mentioning outreach via social media and community organisations to broaden participation.^61^ The intervention in this trial was delivered remotely via an app, whereas most other interventions required face-to-face clinical input.

These pragmatic challenges of trial design can be a barrier to ensuring inclusivity. The two trials that incorporated more detailed plans to include ethnic minority participants also illustrated this. The expansion of recruiting from one NHS Trust in the pilot to 12 Trusts in the full trial and the subsequent removal of the bilingual health advocate perhaps illustrated restrictions within recruiting sites. The authors also acknowledged the pilot trial identified difficulties in managing the number of languages spoken in their intervention, a group model of antenatal care. Therefore, in the full trial, a cap on the number of languages spoken was made.^56^ Although it was clear the trials aimed to embed inclusivity, compromise was still needed to ensure the intervention was feasible, demonstrating the wider considerations required when designing an inclusive trial.

The authors hypothesised that this review would identify an increase in strategies to support the recruitment of ethnic minority participants after 2020, reflecting the publication of the first toolkit for improving the inclusion of Black, Asian and ethnic minority participants in the UK in 2018^35^ followed by the NIHR INCLUDE framework in 2020.^43^ Yet only 4 of the 36 protocols published from 2020 onwards included specific recruitment or retention strategies. This may indicate a lack of awareness or uptake of this guidance, although it may also reflect the time lag from inception of a research project to publication of a protocol. Nonetheless, future considerations should be made as to how guidelines on inclusive research can be effectively implemented. Attaching funding implications may be one solution. For example, the NIHR now stipulates PPIE must be included in grant applications, and 20/33 protocols that referred to PPIE were published from 2020 onwards. The NIHR now stipulates inclusion plans as a condition for funding,^114^ and the Health Research Authority and Medicines and Healthcare products Regulatory Agency are currently piloting a requirement for researchers to submit an Inclusion and Diversity plan with their applications.^115^ Support from funders and regulatory bodies will enforce considerations to be made and ensure more research is developed with inclusion embedded throughout.

Diverse heterogeneity in the ways participants’ ethnicity was reported in trials was identified, which creates difficulties in synthesising evidence and determining representation of participants across trials. Variation of the ONS 5 top level ethnic categories was often used; however, these broad groupings neglect the complexity of ethnicity and make assumptions about the homogeneity of groups.^116^ Although there are no standardised requirements in the reporting of ethnicity, a recently published White Paper from the UK Health Data Research Alliance makes several recommendations of best practices when collecting ethnicity data in research.^117^ Using the 19 core categories from the ONS guidance is advised^101^ to enable comparable reporting across settings. Guidance is also changing in high impact journals including *The Lancet* and *JAMA* on how ethnicity should be collected and reported within research,^118 119^ including recommendations for stating how ethnicity has been defined and operationalised within a study. This was not discussed in any of the papers in this review, and it is recommended future research incorporates this when reporting ethnicity.

When considering diversity and representation in research, the UK Health Data Research Alliance recommends ethnicity should be collected alongside wider determinants of health including socioeconomic status.^117^ This echoes previous recommendations that advised socioeconomic differences should be given equal consideration alongside people’s cultural or genetic factors.^18^ Yet consideration of people’s intersectional identities was not referred to in any of the included papers. This is a barrier in assessing how representative research is. Yet even with this information, assessing representation is complex and context-specific. Across the UK, there is significant variation in ethnicity, deprivation, language and migration across local populations. Therefore, consideration of recruitment strategies may need to be tailored and individualised depending on the place of recruitment. This poses challenges when undertaking large trials conducted across several sites, each with their own form of diversity. Many of the trials included in this review were multicentred, which may have been a barrier to addressing inclusion. Despite these difficulties, Consolidated Standards of Reporting Trials guidance on reporting of RCTs states generalisability of a trial should be included in trial’s discussions,^120 121^ yet many of the results papers did not include this. Determining appropriate representation is relative to the condition under investigation and is not always easy; however, if more information is provided regarding the study population and the population of interest, it will enable the reader to assess both representation of participants and generalisability. This review suggests this is not routinely included, making it difficult to accurately assess the relevance of findings for ethnic minority groups, and raising concerns of whether evidence that is then incorporated into clinical guidelines is generalisable.

### Strengths and limitations

This is the first review to explore how the inclusion of ethnic minority participants is considered within clinical trials in a maternity setting and whether recommendations from inclusion toolkits and frameworks are being incorporated. The combination of published protocols with their results paper provided insights into how inclusive recruitment is considered in research design, what then happens in practice and the outcomes of this. Including trials aimed at recruiting the general population is an additional strength. A previous review on strategies for the recruitment of ethnic minority groups focused on trials that were sampling this population alone;^122^ however, inclusion should be considered in all research to ensure that trials that apply findings to the general population are also appropriately representative.

No comprehensive evaluations of strategies were included in this review; therefore, there were limited findings on successful recruitment or retention approaches in maternity research. However, the aim of this review was to map whether strategies are being used and therefore the search strategy did not aim to identify Studies Within a Trial that focus on the evaluation of recruitment interventions. Exploring previous reviews of evaluated recruitment strategies did not identify any maternity trials in the UK evaluating interventions, however.^123 124^

It is acknowledged this review only reflects trials with published protocols; however, a wide range of maternity subspecialties was represented. It is possible additional recruitment strategies and considerations around representation or inclusion were involved in trial design that were not reported, due to editorial constraints and limits of length of papers. The review also did not undertake a separate search for supplementary discussion papers that may have accompanied studies. In addition, due to the time lag between research conception and publication, we may be yet to see the impact of inclusive research design guidance.

## Conclusions

There is sparse evidence that ethnic representativeness of samples is considered within maternity trials. Few detailed strategies were found to ensure ethnic minority groups are adequately recruited and retained, which may have implications for the generalisability of trials. When ethnic minority groups were included, ethnicity was poorly conceptualised without consideration of the intersectional relationships it embodies.

Toolkits and guidance are available to support the design of inclusive research, but consideration as to how to ensure this is implemented alongside evaluations of their effectiveness is needed. Standardised approaches to collecting and reporting ethnicity should be considered, supported by discussions of the representativeness of samples.

Ethnic inequalities in maternity care have multifactorial causes; however, neglecting to implement steps to ensure those from ethnic minority groups are included and sufficiently reported in research will exacerbate inequitable disparities.

## Supplementary material

10.1136/bmjopen-2025-098926 online supplemental file 1

10.1136/bmjopen-2025-098926 online supplemental file 2

10.1136/bmjopen-2025-098926 online supplemental file 3

## Data Availability

All data relevant to the study are included in the article or uploaded as supplementary information.
