# Supplementary material for: Recruitment, retention and reporting of ethnic representativeness in maternity trials: a scoping review
Source: BMJ Open. 2025 Nov 26;15(11):e098926. doi: 10.1136/bmjopen-2025-098926 (PMC12658542; doi:10.1136/bmjopen-2025-098926)
Supplement: online supplemental file 1 [file bmjopen-15-11-s001.docx]

**Supplementary file 1. Search terms**

Ovid MEDLINE(R) ALL <1946 to March 18, 2024>

1 (embryo* or fetus* or fetus* or fetal* or fetal* or placenta* or transplacenta* or transplacenta* or utero* or uterus* or uterus* or ((preterm or pre-term or premature or term or live or still) adj (birth or births or childbirth* or child-birth* or infant* or newborn* or new-born* or neonate* or baby or babies or labor* or labour* or parturition* or parturient* or delivery or deliveries)) or prematurity or stillbirth* or still-birth* or stillborn* or still-born* or cesarean* or C-section* or abortion* or aborted or miscarr* or birthweight* or birth-weight* or "gestional age" or "gestational hypertension" or eclampsia* or preeclampsia* or pre-eclampsia* or Apgar or ((infant* or newborn* or new-born* or neonate* or baby or babies or mother* or intrauterine or intrauterine) adj1 (mortality or mortalities or dead or death* or loss or lost or demise* or viability))).ti,ab. 1145540

2 (pregnan* or gestation* or fertility or fertile or obstetric* or reproduction or (expectant* adj2 mother*) or "mother-to-be" or "mothers-to-be" or maternit* or maternal or parturient* or "before delivery" or childbearing or child-bearing or gravidit*).ti,ab. 1129562

3 (antenatal* or ante-natal* or antepartum or antepartum or prenatal* or pre-natal* or intrapartum or intrapartum or perinatal* or peri-natal* or neonatal* or neonatal* or postpartum or postpartum).ti,ab. 509556

4 (breastfe* or breast-fe* or breastmilk* or breast-milk* or "breast pumping*" or (milk adj1 (express* or excret* or releas* or secret*)) or lactation or ((human or breast or mother or woman or woman or women or maternal) adj milk*)).ti,ab. 111087

5 Maternal exposure/ or pregnant women/ or exp pregnancy/ or placental circulation/ or breast feeding/ or breast milk expression/ or milk, human/ or congenital abnormalities/ or "prenatal exposure delayed effects"/ 1094758

6 1 or 2 or 3 or 4 or 5 2309488

7 exp Great Britain/ 393491

8 (national health service* or nhs*).ti,ab,in. 286838

9 (english not ((published or publication* or translat* or written or language* or speak* or literature or citation*) adj5 english)).ti,ab. 125333

10 (gb or "g.b." or britain* or (british* not "british columbia") or uk or "u.k." or united kingdom* or (england* not "new england") or northern ireland* or northern irish* or scotland* or scottish* or ((wales or "south wales") not "new south wales") or welsh*).ti,ab,jw,in. 2529965

11 7 or 8 or 9 or 10 2820745

12 (exp africa/ or exp americas/ or exp antarctic regions/ or exp arctic regions/ or exp asia/ or exp oceania/) not (exp great britain/ or europe/) 3398476

13 11 not 12 2664268

14 6 and 13 184197

15 exp Randomized Controlled Trial/ 611895

16 controlled clinical trial.pt. 95588

17 randomized.ab. 638897

18 placebo.ab. 246672

19 drug therapy.fs. 2676925

20 randomly.ab. 429380

21 trial.ab. 689828

22 groups.ab. 2651865

23 15 or 16 or 17 or 18 or 19 or 20 or 21 or 22 5909654

24 exp animals/ not humans.sh. 5204700

25 23 not 24 5168138

26 14 and 25 28445

27 protocol.m_titl. 84952

28 protocol.mp. 461714

29 exp Clinical Trial Protocol/ 12666

30 27 or 28 or 29 461714

31 26 and 30 1328
