# Supplementary material for: Recruitment, retention and reporting of ethnic representativeness in maternity trials: a scoping review
Source: BMJ Open. 2025 Nov 26;15(11):e098926. doi: 10.1136/bmjopen-2025-098926 (PMC12658542; doi:10.1136/bmjopen-2025-098926)
Supplement: online supplemental file 2 [file bmjopen-15-11-s002.docx]

**Supplementary file 2. Data extraction tables**

| **Protocols** |
| --- |
| Study design and research question |
| Sample size |
| Intervention |
| Primary outcome |
| Are the demographics of who should be included within the participants discussed? Is a rationale given? |
| Are specific recruitment strategies planned for ethnic minority participants? |
| What recruitment strategies are planned? |
| Are specific retention strategies planned for ethnic minority participants? |
| What retention strategies are planned? |
| Has PPIE contributed to recruitment and retention strategies? If so, how? |
| Is there evidence of co-production? How? |
| Are their plans to evaluate the strategies? Including quantitative or qualitative process evaluations |
| What are the plans for evaluation? |
| Has intersectionality been considered in any of the recruitment, retention, or evaluations plans? If so, how? |
|  |
| **Results papers** |
| Sample size |
| Participant demographics |
| Is ethnicity reported? If so which ethnic groups were used and how were these determined? |
| Are specific recruitment and retention strategies detailed for ethnic minority participants? |
| If so, do they match the protocol? |
| What recruitment and retention strategies were used for ethnic minority participants? |
| Were strategies evaluated? How were they evaluated? |
| Were qualitative process evaluations undertaken? |
| What were the findings of the evaluations? |
| Is the representativeness of participants included in the findings or discussion? |
| Has intersectionality been considered in discussion or participants or findings? If so, why and how? |
| What conclusions and implications are discussed around representation of participants? |
