# Supplementary material for: Recruitment, retention and reporting of ethnic representativeness in maternity trials: a scoping review
Source: BMJ Open. 2025 Nov 26;15(11):e098926. doi: 10.1136/bmjopen-2025-098926 (PMC12658542; doi:10.1136/bmjopen-2025-098926)
Supplement: Supplementary file 4 [file bmjopen-2025-098926.reviewer_comments.pdf]

PEER REVIEW HISTORY

BMJ Open publishes all reviews undertaken for accepted manuscripts. Reviewers are asked to complete a checklist review form and are provided with free text boxes to elaborate on their assessment. These free text comments are reproduced below.

ARTICLE DETAILS

Title (Provisional)

Recruitment, retention and reporting of ethnic representativeness in maternity trials. A scoping review

Authors

Lovell, Holly; Wicks, Emilie; Thompson, Hilary; Brace, Leanna; Stacey, Tomasina; Rayment-Jones, Hannah; Harding, Seeromanie

---

VERSION 1 - REVIEW

Reviewer 1

Name Sathe, Nila

Affiliation RTI International, Research Triangle Park, NC, USA; RTI International-University of North Carolina Evidence-Based Practice Center US Cochrane Affiliate, Research Triangle Park, NC, USA.

Date 22-Feb-2025

COI None

Thank you for this interesting and timely scoping review. Overall, this review tackles an important issue, uses established methods, and benefits from assessing both published protocols and (where available) results papers. Several sections of the paper should be clarified to improve readers' understanding of the findings. Specifically:

Abstract

-Would benefit from more detail in the Results

Methods

-I believe only RCTs were included but the discussion of including "interventional or RCT study designs" can be interpreted to included non-randomized trials or quasi-experimental

-Clarify how you operationalized the broad questions in your extraction documents; for example, what constituted a "strategy"? Did you have a potential list initially or strategies arose from the extraction? Did the toolkits or recommendations you mention structure the extraction or considerations for reporting results? The Discussion notes considerations of cultural appropriateness; while this is briefly addressed in the results (3 publications

described cultural appropriateness in interventions) it's not clear how this was assessed and if this means appropriateness in the intervention itself or in the recruitment strategies because the discussion of this falls under the Results section re strategies to recruit and retain. This also does not appear explicitly to have been extracted in the extraction tables provided. Some clarification would be useful.

Results

-The presentation of results should be clarified with better framing and additional tables or figures. Some terms need defining to clarify for readers also.

-It is not clear if the team identified 94 or 96 protocols and 65 or 67 results papers (discrepancies between prisma and text)

-It is not explicit how many protocols said nothing about recruitment or retention or representation of ethnic minorities. Presumably this is around 85 protocols, but the opening paragraph of the results should clearly note the Ns—e.g., X% of the 96 (or 94) included protocols reported on recruitment or retention: 8 reported recruitment strategies, 5 included a retention strategy, 2 included both, 4 addressed representation. X% of 67 (or 65) results papers addressed retention: 32 addressed xxx

-Clarify if any results papers discussed recruitment or retention that was not addressed in their corresponding protocols.

-The denominator changes and is not always clear—e.g., 11 papers reported either retention and/or recruitment strategies, but sometime the denominator is 2 (that reported both) or 8 or …

-Clarify if results papers always (presumably) means published RCTs. If so, did you seek companion studies that may have reported on the trial more fully (e.g., you have the protocol but an implementation paper may report on recruitment)?

-If results papers reported subgroup analyses using ethnicity, would that have been captured as an indication of representation?

-Utility of table 1 (which seems to be missing a table line between McCauley and Volkmer) could be improved by integrating the strategies used or other data relevant to this review's specific focus in the table vs. in the figures. Doing so may help you see if there are other inferences possible—e.g., are certain types of interventions (lifestyle, pregnancy care, risk reduction, etc.) more or less likely to describe recruitment approaches in this sample?

-An overall table of key publication characteristics (e.g., elements like year of pub, ethnicities of focus or included, intervention area of focus and Ns of corresponding publications in each category) or a "measles" chart outlining what publications report what elements (i.e., crosswalking the data reported in protocols and results papers with a column of all publication authors/years and rows for the characteristic sought—recruitment, retention, reporting ethnicity characteristics, reporting ethnicity determination, reporting

intersectionality, etc. with checks or stars to indicate if reported). A table or tables like this may help to clarify denominators as well.

Discussion

-paper includes discussion of why this topic and lack and representation is important; several non-UK-focused studies address this topic and may be worth citing and discussing parallels or differnces. For example see Louis-Jacques AF, Heuberger AJ, Mestre CT, Evans VF, Wilson RE, Gurka MJ, Lewis TR. Improving Racial and Ethnic Equity in Clinical Trials Enrolling Pregnant and Lactating Individuals. J Clin Pharmacol. 2023 Jun;63 Suppl 1:S21-S33. doi: 10.1002/jcph.2263. PMID: 37317498.

-Is not searching the ISRCTN, which can be limited to studies with protocols, a limitation?

-If the relevant toolkits and guidance cited report strategies specific to ethnic minorities , it may be helpful to note in more detail explicitly what was/was not addressed in the literature you identified, though you've clearly noted that the reporting is limited, and you have noted PPIE as an example of a recommended technique.

Overall

-Minor copyediting needed (subject verb agreement and tenses, which vs. that, etc.)

-Clarify some text—e.g., pg. 3, lines 7-8 does this mean deaths occurring in Black and Asian birthing people outside the UK?

-When using terms like "poorer maternal outcomes" clarify poorer than what referent.

**Reviewer**	**2**
**Name**	**Bengesai, Annah V**
**Affiliation**	**University of KwaZulu-Natal**
**Date**	**02-Sep-2025**
**COI**	**None**

Thank you for the opportunity to review this manuscript. Overall, it is well-written and addresses a relevant research question regarding health equity in maternity care. However, some areas could be enhanced as outlined below:

Background

The background is well written and justifies the need for this research. However, a more critical examination of the systemic factors responsible for addressing the inequalities would be beneficial. Also, given that guidelines and toolkits exist to facilitate inclusive research design, a more in-depth exploration of why representation remains problematic would strengthen the argument.

It is also unclear what is meant by appropriate representation, and it would be beneficial to operationalise this term as it pertains to the study.

Furthermore, no justification is given as to why a scoping review method was chosen over other systematic review approaches, or why the period 2004-2024 was chosen.

Findings

I am concerned that missing information is interpreted as evidence of poor practice or lack of commitment, without considering alternative explanations as to why its missing, such as editorial constraints, lack of space or publication in supplementary materials.

The observation that strategies are mostly 'brief references' requires substantiation- for instance, what are the defining characteristics of these brief references? How would one differentiate between a brief reference and detailed plans?

Did the studies documenting PPIE involvement achieve better participant representation than those without?

Overall, the current analysis reads more like a descriptive summary than a critical review that advances understanding of inclusive research practices.

VERSION 1 - AUTHOR RESPONSE

Reviewer 1

Comments to the Author:
Thank you for this interesting and timely scoping review. Overall, this review tackles an important issue, uses established methods, and benefits from assessing both published protocols and (where available) results papers. Several sections of the paper should be clarified to improve readers' understanding of the findings. Specifically:

Abstract
-Would benefit from more detail in the Results

Added within the constraints of the word limit

Methods
-I believe only RCTs were included but the discussion of including "interventional or RCT study designs" can be interpreted to included non-randomized trials or quasi-experimental

Edited and removed "interventional study designs" to clarify only RCTs were included

-Clarify how you operationalized the broad questions in your extraction documents; for example, what constituted a "strategy"? Did you have a potential list initially or

strategies arose from the extraction? Did the toolkits or recommendations you mention structure the extraction or considerations for reporting results?

Thank you, we have added this under data extraction:

Recruitment and retention strategies were broadly defined, to include any research design consideration that specifically referred to consideration of ethnic minority populations, inclusion or representation. Extracted statements could range from a single sentence to a broader description. Identification was based on knowledge of recommendations from inclusion frameworks and the authors expertise in trial conduct.

The Discussion notes considerations of cultural appropriateness; while this is briefly addressed in the results (3 publications described cultural appropriateness in interventions) it's not clear how this was assessed and if this means appropriateness in the intervention itself or in the recruitment strategies because the discussion of this falls under the Results section re strategies to recruit and retain. This also does not appear explicitly to have been extracted in the extraction tables provided. Some clarification would be useful.

Thank you, we have elaborated on this in the results section to make it clearer. It was included within the "planned retention strategies" extraction column. Cultural appropriateness was not on the extraction template, and instead emerged as a theme under retention strategies. I have edited the results section to clarify this:

Three papers stated they considered cultural needs and socio-economic circumstances in the design of their interventions, which may support retention of ethnic minority participants[62-64]. This included integrating cultural preferences when giving dietary advise in a trial on postnatal weight management in pregnancy [62] and culturally adaptable recipes in a trial examining the impact of a Mediterranean style diet on outcomes for women with metabolic risk factors [64].

Results
-The presentation of results should be clarified with better framing and additional tables or figures. Some terms need defining to clarify for readers also.

Thank you, we have responded to this further below regarding your suggestions with the tables.

-It is not clear if the team identified 94 or 96 protocols and 65 or 67 results papers (discrepancies between prisma and text)

Thank you for highlighting. This error occurred when updating the search. I have corrected these discrepancies.

-It is not explicit how many protocols said nothing about recruitment or retention or representation of ethnic minorities. Presumably this is around 85 protocols, but the opening paragraph of the results should clearly note the Ns—e.g., X% of the 96 (or 94) included protocols reported on recruitment or retention: 8 reported recruitment strategies, 5 included a retention strategy, 2 included both, 4 addressed representation. X% of 67 (or 65) results papers addressed retention: 32 addressed xxx

Thank you for this suggestion. We have added to the results section how many protocols did not include strategies. We have not included all the numbers in the opening paragraph to avoid repetition where we have included them under each review question.

-Clarify if any results papers discussed recruitment or retention that was not addressed in their corresponding protocols.

No results papers discussed strategies that were not addressed in their protocols. We have added this to the results section.

-The denominator changes and is not always clear—e.g., 11 papers reported either retention and/or recruitment strategies, but sometime the denominator is 2 (that reported both) or 8 or ...

Thank you, for highlighting this. The denominators are correct, but we have made some changes to make this clearer where it does change.

-Clarify if results papers always (presumably) means published RCTs.

We have clarified this in the types of sources section:

Available papers reporting results of RCTs of the included protocols that met the inclusion criteria were also included.

If so, did you seek companion studies that may have reported on the trial more fully (e.g., you have the protocol but an implementation paper may report on recruitment)?

No, we didn't undertake an additional search to identify companion papers, although where a paper referred to a companion paper we did read this but did not identify any additional findings related to ethnicity.

-If results papers reported subgroup analyses using ethnicity, would that have been captured as an indication of representation?

6 papers did include this, but it wasn't discussed whether the sample sizes were powered for this to be significant. We don't believe it was therefore indicative of considering representation, rather it just included with other demographics at looking at differences in findings. We have added this comment into the discussion section:

It may be argued including a sub-group analysis by ethnic group is an indicator of consideration of representation. Although 6 papers did include this, it was not discussed whether sample sizes were powered for this to be significant. Therefore, subgroup analysis in the absence of discussion around adequately powered sample sizes for each group is not an indication of representation.

-Utility of table 1 (which seems to be missing a table line between McCauley and Volkmer) could be improved by integrating the strategies used or other data relevant to this review's specific focus in the table vs. in the figures. Doing so may help you see if there are other inferences possible—e.g., are certain types of interventions (lifestyle, pregnancy care, risk reduction, etc.) more or less likely to describe recruitment approaches in this sample?
-An overall table of key publication characteristics (e.g., elements like year of pub, ethnicities of focus or included, intervention area of focus and Ns of corresponding publications in each category) or a "measles" chart outlining what publications report what elements (i.e., crosswalking the data reported in protocols and results papers with a column of all publication authors/years and rows for the characteristic sought—recruitment, retention, reporting ethnicity characteristics, reporting ethnicity determination, reporting intersectionality, etc. with checks or stars to indicate if reported). A table or tables like this may help to clarify denominators as well.

Thank you for these useful suggestions. We have reconsidered the utility of table one, and moved it to the supplementary files. We have removed the figures and added 2 additional tables. Table 1 provides an overview of what was included within both protocols and RCT results papers in relation to the review questions. Table 2 uses checks to illustrate which strategies were included in which protocols, whilst also highlighting the intervention.

Discussion
-paper includes discussion of why this topic and lack and representation is important; several non-UK-focused studies address this topic and may be worth citing and discussing parallels or differnces. For example see Louis-Jacques AF, Heuberger AJ, Mestre CT, Evans VF, Wilson RE, Gurka MJ, Lewis TR. Improving Racial and Ethnic Equity

in Clinical Trials Enrolling Pregnant and Lactating Individuals. J Clin Pharmacol. 2023 Jun;63 Suppl 1:S21-S33. doi: 10.1002/jcph.2263. PMID: 37317498.

Thank you for signposting to this interesting paper. We have incorporated this and more details on this topic into the discussion:

Failing to ensure those who experience the poorest outcomes are included in research can exacerbate inequalities. This is due to reduced generalisability of trials, missing differences in responses to interventions, fuelling lack of trust. The role of under-representation in research perpetuating inequalities has also been demonstrated to have an economic impact [109].

A range of reasons for under-representation of ethnic minority groups in research are discussed in the literature. Barriers to participation include restrictive study designs, poor engagement with communities, and a history of mistrust, with most evidence from the USA and the UK and no substantial differences reported between the two countries [35, 37, 110, 111]

-Is not searching the ISRCTN, which can be limited to studies with protocols, a limitation?

When designing the protocol for the review we explored searching the ISRCTN for protocols, however it doesn't provide a function to search by study design, i.e. RCTs and therefore the decision was made to use the included databases.

-If the relevant toolkits and guidance cited report strategies specific to ethnic minorities , it may be helpful to note in more detail explicitly what was/was not addressed in the literature you identified, though you've clearly noted that the reporting is limited, and you have noted PPIE as an example of a recommended technique.

Thank you, we have now expanded on this in the discussion section:

To address these issues a range of recommendations have been published to support the inclusion of ethnic minority participants across the research cycle [35, 37, 42-44]. These include identification of who might be under-represented within the researcher's population of interest and who experiences higher burden of disease, and then exploring potential barriers to their inclusion and how they might be mitigated [43]. This may involve designing different formats and methods of sharing study information and working with communities to identify appropriate and flexible recruitment pathways [35, 37, 44, 111]. No protocols provided substantiative discussions that suggested the demographics of who should be included had been considered at the design stage and incorporated into recruitment strategies.

Overall

-Minor copyediting needed (subject verb agreement and tenses, which vs. that, etc.)

-Clarify some text—e.g., pg. 3, lines 7-8 does this mean deaths occurring in Black and Asian birthing people outside the UK?

Edited

-When using terms like "poorer maternal outcomes" clarify poorer than what referent.

Added:

Black and Asian women experience significantly higher rates of mortality and morbidity perinatally and are more likely to lose their babies

Reviewer: 2

Thank you for the opportunity to review this manuscript. Overall, it is well-written and addresses a relevant research question regarding health equity in maternity care. However, some areas could be enhanced as outlined below:

Background

The background is well written and justifies the need for this research. However, a more critical examination of the systemic factors responsible for addressing the inequalities would be beneficial.

Thank you, we have expanded upon this as much is possible within the word limits:

Ethnic health inequalities are shaped by multifactorial influences, often explained through the structural determinants of health such as housing, education, and employment [19]. Area deprivation, an ecological correlate of these determinants, is strongly associated with adverse maternal outcomes; women and babies in the most deprived areas face the highest mortality risks [8]. Recent evidence from the UK national population-based cohort study showed that women of Black ethnicity remained at significantly increased risk of maternal death compared with women of White ethnicity after adjustments for area deprivation [20]. Reviews have also highlighted that persistent inequalities reflect the impact of structural racism, implicit bias, and discriminatory practices within maternity care. [21-28]. Underrepresentation of ethnic minority groups in research, inadequate recognition of cultural and linguistic needs, and reliance on Eurocentric models of care can further exacerbate these disparities, perpetuating mistrust, disengagement, and poorer health outcomes for minority groups.

An intersectional perspective is essential to fully interrogate these inequalities. Ethnicity does not operate in isolation but intersects with gender, class, migration status, and other

axes of structural disadvantage to shape women's experiences of maternity care [29, 30]. Ignoring these intersections risks oversimplifying the mechanisms driving inequality and overlooking groups who experience multiple disadvantage [31, 32]. Addressing ethnic health inequalities therefore requires structural reform: embedding accountability for equity within healthcare systems, developing culturally safe and inclusive models of care, and diversifying research participation to ensure the complexity of intersecting inequalities is adequately captured.

Also, given that guidelines and toolkits exist to facilitate inclusive research design, a more in-depth exploration of why representation remains problematic would strengthen the argument.

Thank you. We have added this:

The available recommendations are not mandated and have not been evaluated. Therefore, it is unknown the extent to which they are utilised, how many researchers are aware of them, or what their impact is.

It is also unclear what is meant by appropriate representation, and it would be beneficial to operationalise this term as it pertains to the study.

Thank you, we have added this into the introduction and also a reflection of this into the discussion:

Representation can often be difficult to assess, and it is dependent on factors including where the research was conducted, the condition under examination, and who the results will be applied to. Therefore, the term "appropriate representation" may be more useful when describing representation to acknowledge that comparing study populations to the general population is not always suitable.

Furthermore, no justification is given as to why a scoping review method was chosen over other systematic review approaches, or why the period 2004-2024 was chosen.
Added in the methods section:

A 20-year period was searched, as representation in research is a relatively recent priority, therefore it was unlikely older research would include detailed considerations on the topic.

Findings
I am concerned that missing information is interpreted as evidence of poor practice or lack of commitment, without considering alternative explanations as to why its missing,

such as editorial constraints, lack of space or publication in supplementary materials. Thank you, we have expanded the section in the limitations which discusses this and also made reference to this in the abstract by adding that few maternity trials "report" considerations around representation.

The observation that strategies are mostly 'brief references' requires substantiation- for instance, what are the defining characteristics of these brief references? How would one differentiate between a brief reference and detailed plans?

Thank you, we have provided additional clarification in the discussion section:

Despite available guidance, , only two of the 11 protocols that considered specific recruitment or retention strategies detailed comprehensive approaches [56, 57] the remaining papers providing a brief reference to considerations made. These were often single sentences stating, for example, that sites with diverse populations would be chosen, or that an interpreter would be used where required. Protocols which reported more detailed considerations gave descriptions as to the different ways language needs would be supported, alongside discussion as to why inclusive approaches were important.

Did the studies documenting PPIE involvement achieve better participant representation than those without?

No, apparent representation was very varied in the papers reporting PPIE. We have not included this in the paper as it would require more exploration as to what kind of PPIE was undertaken, who was involved, and how, to explore the impact of PPIE on representation. This level of information was not included within the protocols.

Overall, the current analysis reads more like a descriptive summary than a critical review that advances understanding of inclusive research practices.

Thank you for your feedback, we have edited this section accordingly.

VERSION 2 - REVIEW

Reviewer 1

Name Sathe, Nila

Affiliation RTI International, Research Triangle Park, NC, USA; RTI International-University of North Carolina Evidence-Based Practice Center US Cochrane Affiliate, Research Triangle Park, NC, USA.

Date **26-Oct-2025**

COI

Thanks for your thoughtful revisions to this manuscript. Overall, the paper is clearer but could benefit from a few minor revisions to improve clarity for readers:

-Suggest limiting use of passive voice unless that is the journal style

-Additional clarity on how you operationalized extraction:

• I appreciate the clarification of how you extracted statements about recruitment or representation; however, I think an example may be useful readers to contextualize your approach from the outset—e.g.,

… to include any research design consideration that specifically referred to consideration of ethnic minority populations, inclusion or representation. Extracted statements could range from a single sentence to a broader description (e.g., protocol reports specific recruitment materials for an ethnic group).

• In addition, while you've cited inclusion frameworks and trial expertise, it may be useful to provide an example here also to contextualize (e.g. ..Identification was based on knowledge of recommendations from inclusion frameworks (e.g., SPIRIT reporting guidelines require….) and the authors expertise in trial conduct.

-You comment on temporal trends in the literature you identified; did you see any trends by study area or intervention type (e.g., behavioral, drug, early pregnancy, post-partum) that may be relevant to call out?

-Per the prisma diagram, there should be 95 (139-44) protocols but text references 96.

-Thanks for adding the appendix table; however, the presentation of it is unclear as the rows break across pages, and it's not clear what data pertains to what paper. Suggest revising the presentation or including an excel file of the data

-You edited based on this earlier comment but the meaning is still not clear—poor outcomes are in comparison to White/majority population?

When using terms like "poorer maternal outcomes" clarify poorer than what referent.

Added: Black and Asian women experience significantly higher rates of mortality and morbidity perinatally and are more likely to lose their babies

---

VERSION 2 - AUTHOR RESPONSE

Reviewer: 1
Dr. Nila Sathe, RTI International, Research Triangle Park, NC, USA; RTI International-University of North Carolina Evidence-Based Practice Center US Cochrane Affiliate, Research Triangle Park, NC, USA.

Comments to the Author:
Thanks for your thoughtful revisions to this manuscript. Overall, the paper is clearer but could benefit from a few minor revisions to improve clarity for readers:
-Suggest limiting use of passive voice unless that is the journal style

Thank you for all your comments to date. We have edited throughout to remove the passive voice where possible.

-Additional clarity on how you operationalized extraction:
• I appreciate the clarification of how you extracted statements about recruitment or representation; however, I think an example may be useful readers to contextualize your approach from the outset—e.g.,
... to include any research design consideration that specifically referred to consideration of ethnic minority populations, inclusion or representation. Extracted statements could range from a single sentence to a broader description (e.g., protocol reports specific recruitment materials for an ethnic group).

Thank you for this suggestion. We have added: for example a protocol may state information is translated to increase participation of those from ethnic minorities.

• In addition, while you've cited inclusion frameworks and trial expertise, it may be useful to provide an example here also to contextualize (e.g. ..Identification was based on knowledge of recommendations from inclusion frameworks (e.g., SPIRIT reporting guidelines require....) and the authors expertise in trial conduct.

Thank you, we have added an example: for example the NIHR INCLUDE ethnicity framework recommends researchers identify which populations should be included in the trial, and then designing targeted recruitment methods to ensure it is accessible to those groups.

-You comment on temporal trends in the literature you identified; did you see any trends by study area or intervention type (e.g., behavioral, drug, early pregnancy, post-partum) that may be relevant to call out?

No, they didn't appear to be trends in terms of when the pregnancy trajectory the research was conducted, however it was noted that interventions that we behaviour were more likely to be culturally adapted. We mentioned this in the discussion:

The design of interventions should be culturally appropriate to support recruitment and retention[37], however only three trials incorporated these considerations [62-64]. It may be that some interventions lend themselves to being more culturally adaptable

than others, as these trials were testing either a behavioural or dietary intervention, as opposed to medication or a device.

-Per the prisma diagram, there should be 95 (139-44) protocols but text references 96.

Thank you for highlighting this. We have corrected the Prisma diagram as the total number of studies excluded was calculated incorrectly, it should have totalled 43.

-Thanks for adding the appendix table; however, the presentation of it is unclear as the rows break across pages, and it's not clear what data pertains to what paper. Suggest revising the presentation or including an excel file of the data

Apologies the presentation is not clear. We uploaded the supplementary table as an Excel file, however I am uncertain in what format this was then forwarded on. I will request that it is uploaded as an Excel file once published.

-You edited based on this earlier comment but the meaning is still not clear—poor outcomes are in comparison to White/majority population?
When using terms like "poorer maternal outcomes" clarify poorer than what referent.
Added: Black and Asian women experience significantly higher rates of mortality and morbidity perinatally and are more likely to lose their babies

Thank you, we have added "compared to White women" in both the abstract and the introduction to clarify this.
